# Towards an integrated primary and secondary HIV prevention continuum for the United States: a cyclical process model

**DOI:** 10.7448/IAS.19.1.21263

**Published:** 2016-11-17

**Authors:** Tim Horn, Jennifer Sherwood, Robert H Remien, Denis Nash, Judith D. Auerbach, for the Treatment Action Group and Foundation for Aids Research HIV Prevention Continuum Working Group

**Affiliations:** 1 Treatment Action Group, New York, NY, USA; 2 amfAR, the Foundation for AIDS Research, Washington, DC, USA; 3 HIV Center for Clinical and Behavioral Studies, Columbia University, New York, NY, USA; 4 Institute for Implementation Science in Population Health, School of Public Health, City University of New York, New York, NY, USA; 5 Center for AIDS Prevention Studies, School of Medicine, University of California, San Francisco, CA, USA

**Keywords:** HIV, prevention, continuum, PrEP, process model, cycle, testing

## Abstract

**Introduction:**

Every new HIV infection is preventable and every HIV-related death is avoidable. As many jurisdictions around the world endeavour to end HIV as an epidemic, missed HIV prevention and treatment opportunities must be regarded as public health emergencies, and efforts to quickly fill gaps in service provision for all people living with and vulnerable to HIV infection must be prioritized.

**Discussion:**

We present a novel, comprehensive, primary and secondary HIV prevention continuum model for the United States as a conceptual framework to identify key steps in reducing HIV incidence and improving health outcomes among those vulnerable to, as well as those living with, HIV infection. We further discuss potential approaches to address gaps in data required for programme planning, implementation and evaluation across the elements of the HIV prevention continuum.

**Conclusions:**

Our model conceptualizes opportunities to monitor and quantify primary HIV prevention efforts and, importantly, illustrates the interplay between an outcomes-oriented primary HIV prevention process and the HIV care continuum to move aggressively forward in reaching ambitious reductions in HIV incidence. To optimize the utility of this outcomes-oriented HIV prevention continuum, a key gap to be addressed includes the creation and increased coordination of data relevant to HIV prevention across sectors.

## Introduction

The HIV care continuum has become a highly visual, accessible and reproducible model to improve health outcomes and minimize transmission risk among those living with the virus [1]. Viral load suppression is viewed not only as the continuum's central outcome to minimize individual morbidity and mortality, but also as a key intervention for secondary HIV prevention, given that viral suppression reduces the risk of HIV transmission [2]. Reducing the risk of HIV acquisition among those not already infected and vulnerable to HIV exposure is equally essential.

An HIV prevention continuum, like the care continuum, is potentially valuable to identify opportunities at key steps in an HIV incidence- and health outcomes-oriented process. Such a model affords the opportunity to: 1) define best biomedical, behavioural and ancillary support practices, including those that foster integration of HIV prevention with broader primary care, wellness promotion and sexual and reproductive health services; 2) further articulate and refine the metrics of success; 3) identify gaps in provider/intervention access and utilization; 4) inform the allocation of human and financial resources; 5) establish implementation science priorities; and 6) generate and support advocacy for the highest impact HIV prevention activities.

Primary HIV prevention continua and similar heuristics have been developed by others. These include a generalized, population-based approach [3]; an infection cascade and prevention pathways model [4]; and pre-exposure prophylaxis (PrEP) and other intervention-specific cascades [5–7]. Proposed models are not without limitations, however. Most fundamentally, unlike engagement in specialized care and antiretroviral therapy after an HIV diagnosis, initiation of a particular intervention following an HIV-negative diagnosis is neither routine nor straightforward. HIV prevention needs and options are not universal or static because of individual and intrapopulation variability and temporal fluctuations in risk. Additionally, few proposed models address congruity with the HIV care continuum. A heuristic device illustrating the importance of both primary and secondary HIV prevention may prove useful in further influencing HIV incidence.

Recognizing inherent challenges, we present a novel continuum model for the United States as a conceptual framework for addressing individualized primary HIV prevention needs to achieve population-level reductions in HIV acquisition risk and to illustrate the critical link between a comprehensive primary prevention process and the care continuum to further improve health outcomes and minimize transmission risk among those who are infected with HIV. To bolster stakeholder interest in this model, particularly among U.S. public health departments, we discuss potential approaches to address gaps in data required for programme planning, implementation, and evaluation across the elements of the primary HIV prevention continuum.

## Discussion

**Figure 1:**
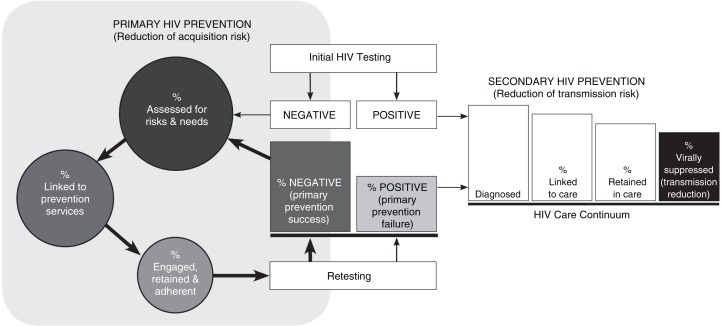
Comprehensive HIV prevention processes. Conceptual framework illustrating the interplay between processes to halt both the acquisition and transmission of HIV. The primary HIV prevention cycle, left, begins with HIV testing. Risk and needs assessments, linkage to services, engagement in risk-reduction prevention interventions and HIV testing are repeated for as long as an individual remains at risk for HIV acquisition.

Our model, shown in Figure 1, configures the primary HIV prevention continuum as a cycle, recognizing that the primary goal of remaining HIV negative, confirmed by repeat testing, is not a static process but rather a dynamic one, dependent on population, network and individual fluctuations in biomedical and supportive care needs over time. For example, not all HIV-negative gay, bisexual and other men who have sex with men will necessarily benefit from the HIV protection afforded by PrEP, including some men in sexually exclusive relationships and men who use condoms consistently and effectively. Even among those for whom PrEP is indicated and desired, utilization may be limited due to transient periods of risk.

A unique feature of the proposed model is a depiction of the link between the primary HIV prevention continuum and the HIV care continuum and the critical interplay of both in maximizing health outcomes and minimizing ongoing spread of the virus. For example, an HIV-negative individual who seroconverts while engaged in a prevention programme featuring regular HIV testing may quickly be linked to and engaged in HIV care and, thereby, benefit from early antiretroviral therapy, including reduction in further onward HIV transmission.

### HIV testing and retesting

HIV testing is the entry point in the HIV prevention cycle, as it generally provides a critical point of contact with the healthcare and service delivery systems for individuals who are HIV negative but are vulnerable to the infection, as well as being a gateway to treatment for people diagnosed with HIV infection. The cycle is repeated for as long as an individual remains at risk for HIV acquisition. In effect, 100% of people at risk for HIV infection who test seronegative at the beginning and end of one cycle should re-enter the cycle and be retested at least annually [8–10], coupled with regular HIV prevention risk and needs assessments to determine linkage and intervention needs.

### Risk and needs assessment

As part of a comprehensive prevention strategy, following a negative HIV test, people vulnerable to HIV infection should receive risk and needs assessments. These can best ensure linkage to medical and other service providers capable of providing or coordinating evidence-based interventions and social services appropriate for that individual. The cyclical framework of our model, with HIV retesting as a central indicator, allows for repeated risk and needs assessments to meet an individual's changing HIV prevention requirements.

For those testing for HIV in acute care (e.g. emergency departments) or non-healthcare settings (e.g. community-based organizations (CBOs)), minimum assessments may include knowledge of and eligibility for a range of risk-reduction strategies, including PrEP, in accordance with U.S. Centers for Disease Control and Prevention (CDC) recommendations, and healthcare provider and insurance status navigation needs [11]. For vulnerable individuals testing for HIV in healthcare settings, or having been referred for HIV prevention services by an acute care or non-healthcare testing site, possible assessments include the following: screenings for sexually transmitted infections, mental health disorders, substance abuse, intimate partner violence and trauma; adequacy of health insurance to cover necessary prevention services; and other primary or specialty care needs [12,13]. The development of simple risk and needs assessment instruments, including online self-assessment and navigation tools, to help maximize linkage to and engagement in HIV prevention services while minimizing the added burden to providers and systems should be an implementation priority.

### Linkage to prevention services

For individuals tested for HIV and having undergone comprehensive risk and needs assessments in primary care settings, linkage may include referrals to specialized medical, mental health and substance abuse services and CBO-provided psychosocial and ancillary services (e.g. housing, employment, nutritional and social support) [12,13]. For individuals tested for HIV and having undergone basic risk assessments in acute and non-healthcare settings, linkage to culturally sensitive medical and other service providers with knowledge and experience providing or coordinating various primary HIV prevention interventions is essential [11]. These may include providers in primary care settings; reproductive, sexual, transgender or other community health programmes (including PrEP clinics); and intervention programmes at CBOs. In all cases, assistance with health insurance and other benefits, including linkage to health insurance navigators, case management and intervention-specific programmes (e.g. PrEP medication and co-pay assistance programmes), must be prioritized to ensure adequate coverage for medical care and other services identified as necessary during risk and needs assessments.

### Engagement, retention and adherence

The final element in the primary prevention cycle addresses uptake of, engagement in and adherence to optimal, high quality HIV prevention and risk-reduction interventions. These include PrEP; post-exposure prophylaxis (PEP); syringe exchange programmes (SEPs) and substance abuse treatment; mental health services; housing assistance; sexual health services; and behavioural change interventions. To help optimize engagement in HIV prevention programmes and related systems of care, the use of culturally competent case managers, patient navigators and/or other client-centred services should be considered [14–18].

### Obtaining essential data

In contrast to the relatively straightforward data elements used to assess outcomes along the HIV care continuum, the metrics required to populate a primary HIV prevention continuum involving different systems of service delivery, interventions and outcome measures are incredibly complex and often without adequate or complete population-based data sources. Here we discuss two potential approaches to address the gaps in data required for programmatic planning, implementation and evaluation across the elements of the prevention cycle.

#### Primary HIV prevention continuum metrics

One strategy involves coordinating existing HIV prevention-related data. For example, the U.S. CDC, utilizing data from the National Health and Nutrition Examination Survey, the National Survey on Drug Use and Health and the National Survey of Family Growth, has estimated the number of U.S. adults with indications for PrEP based on behavioural risk factors for HIV and the 2014 U.S. Public Health Service's PrEP clinical practice guideline to be 1,232,000 (95% confidence interval: 661,000 to 1,803,000) [19,20]. This effort to organize and analyze data across national surveys produced a valuable estimate of the number of US adults at elevated HIV risk, which could be used immediately as a lower-bound threshold for the prevention cycle's primary denominator, as well as to evaluate PrEP coverage and advocate for scale-up.

A similar large-scale effort to identify and validate data sources for mid-cycle elements of the HIV prevention continuum, such as rates of health insurance coverage, linkage to service providers and utilization of evidence-based interventions, has not yet been undertaken. One potential data source for this work might be the National HIV Prevention Program Monitoring and Evaluation (NHM&E) system, which collects current and prior HIV testing data, referrals to specific HIV prevention activities and “intervention completion” each time a client enrols in or completes an intervention after HIV testing at a CDC directly funded CBO [21]. Available publications, presentations and reports from NHM&E data focus primarily on HIV testing results [22–24]. Collating and publishing data on referrals and completion of HIV prevention services, as well as indication for HIV testing, would be an important contribution – albeit a labour-intensive one – to informing HIV prevention service coverage and building local or national HIV prevention continua. Other potential data sources to inform elements of the HIV prevention cycle are listed in Table 1.

**Table 1:** Potential elements, metrics and data sources for the primary HIV prevention cycle

Step	HIV testing and retesting	Risk and needs assessment	Linkage to prevention services	Engagement, retention and adherence
Elements and metrics	Testing through: community health centres; physician offices; hospital-based inpatient and ambulatory care clinics; emergency departments; CBO/ASO; home/self-testing; harm reduction and substance use programmes; mobile/venue-based units	STI screening; pregnancy and family planning; mental health and substance abuse; trauma and violence; insurance coverage; primary care engagement; housing and employment status; and sexual health screenings	Documented linkage to: health insurance, including ACA/health insurance navigation; primary care provider or community-based PrEP or PEP providers; syringe exchange and other harm reduction programmes; and/or DIS/public health departments	Engagement (number/type of visits); client-provider relationship; intervention adherence (e.g. uptake and continued utilization of PrEP and PEP)
Data sources	NHM&E; health departments; community clinics; labs (public and private); ACA plans; CMS and state Medicaid databases; Veterans Administration health centres; prisons and jails; Bureau of Primary Health Care/HRSA; ob-gyn; emergency rooms	ICD 9 and 10; CBO programmatic and client data; Healthy People 2020	NHM&E; additional data sources needed	NHM&E, BRFSS, YRBS, NHBS, NSFG, PRAMS, CMS, and MMP hospital discharge data; data brokers; Medicaid registries; and CBO programme data, including housing and supportive services

ACA, the Patient Protection and Affordable Care Act; ASO, AIDS service organization; BRFSS, Behavioral Risk Factor Surveillance System; CBO, community-based organization; CMS, the Centers for Medicare and Medicaid Services; DIS, disease intervention specialists; HRSA, Health Resources and Services Administration; MMP, Medical Monitoring Project; NHBS, National HIV Behavioral Surveillance; NHM&E, National HIV Prevention Monitoring and Evaluation; NSFG, National Survey of Family Growth; PEP, post-exposure prophylaxis; PRAMS, Pregnancy Risk Assessment Monitoring System; PrEP, pre-exposure prophylaxis; STI, sexually transmitted infection; YRBS, Youth Risk Behavior Surveillance System.

#### Identifying “missed opportunities” and primary HIV prevention continuum gaps

A second approach includes leveraging extant, robust HIV surveillance data among individuals who tested positive for the virus. Since the early days of the U.S. epidemic, such data pertaining to transmission risk among individuals testing positive for HIV have been used to inform HIV prevention planning and funding.

Each new infection continues to represent a missed opportunity for primary HIV prevention. In the setting of expanded prevention options available to those vulnerable to HIV infection, there is a need for renewed and strategic use of HIV surveillance data on new diagnoses to systematically understand prevention gaps and missed prevention opportunities, with rapid translation to “reverse engineer” primary HIV prevention continuum element priorities. Other areas of public health, including efforts to prevent maternal mortality, have employed similar approaches and have benefited from studying “near misses” in order to inform population-level best practices and implementation strategies [25–27].

Current CDC data systems allow for the collection of information to make some inferences about an individual's attributable risk factor(s) for HIV acquisition and to track linkages to appropriate services after receiving a positive diagnosis. However, not enough surveillance data are gathered following diagnosis to learn about missed HIV prevention opportunities inherent in every new HIV infection. Treating each new infection as an sentinel health event is necessary to understand exactly where gaps in the primary HIV prevention continuum are occurring, especially as PrEP and PEP implementation is ramping up in many jurisdictions. Examples of probable gaps include the following: lack of knowledge regarding the symptoms of, and high transmission risk during, acute HIV infection; lack of awareness or availability of PrEP, PEP, SEP or other interventions; poor retention in or adherence to prevention services; and structural barriers to affordable health insurance, adequate medical care, safe housing or other supportive services. A fundamental assumption in the reverse engineering approach is that people vulnerable to HIV infection will have similar characteristics and risk factors to those who were recently diagnosed, such that extrapolation of data between populations is valid. Local programmes targeted at increasing early or immediate treatment following HIV testing, such as the University of California, San Francisco's RAPID programme [28], are well poised to collect data on recently diagnosed individuals and inform data for local HIV prevention gaps.

## Conclusions

Our proposed model provides a standardized roadmap for moving aggressively forward in reaching national HIV incidence and care targets by: 1) conceptualizing primary HIV prevention as a repeating, comprehensive and outcomes-oriented process that is applicable to vulnerable populations comprised of individuals with fluctuating risks and intervention needs; and 2) illustrating the value of HIV retesting as both a metric to continuously gauge the effectiveness of the primary HIV prevention cycle and an opportunity to streamline successful linkage, engagement and viral load suppression through the HIV care continuum. A limitation of our model is the absence of the complete data required to test the proposed primary HIV prevention continuum steps, which will be necessary indicators of success. An obstacle to model validation is the lack of a robust and coordinated HIV prevention monitoring system, which is sorely needed to help guide the implementation of key modalities of HIV prevention, such as PrEP and PEP. Our hope is that, in suggesting a way forward, we can catalyze an effort to address these limitations, which have stymied the promulgation of a meaningful primary HIV prevention continuum to date.

Given the current state of data collection infrastructure in the United States, efforts to vitalize HIV prevention will need to focus on increasing coordination between existing systems, including those not specifically focused on HIV, to prioritize the reporting of data for people vulnerable to HIV infection. The need for these data, however, must be weighed against efforts to decrease federal data collection and reporting burdens among health departments and CBOs as well as the need for increased resources required to more fully support these surveillance activities. Creating new primary prevention-focused variables in existing data collection tools, as well as conducting more analyses of current variables, will inform regional, state and national public health evaluation of prevention service coverage, identify gaps and facilitate advocacy for scale-up of highly effective prevention services in the United States.
